# Inositol hexakisphosphate primes syndapin I/PACSIN 1 activation in endocytosis

**DOI:** 10.1007/s00018-022-04305-2

**Published:** 2022-05-09

**Authors:** Yue Shi, Kaixuan Zhao, Guang Yang, Jia Yu, Yuxin Li, Michael M. Kessels, Lina Yu, Britta Qualmann, Per-Olof Berggren, Shao-Nian Yang

**Affiliations:** 1 The Rolf Luft Research Center for Diabetes and Endocrinology, Karolinska Institutet, Karolinska University Hospital L1, 171 76 Stockholm, Sweden; 2 Jilin Academy of Traditional Chinese Medicine, Changchun, 130021 China; 3 National Engineering Laboratory for Druggable Gene and Protein Screening, Northeast Normal University, Changchun, 130024 China; 4 Institute for Biochemistry I, Jena University Hospital/Friedrich Schiller University Jena, 07743 Jena, Germany; 5 Center for Diabetes and Metabolism Research, Division of Endocrinology and Metabolism, West China Hospital, Sichuan University, Chengdu, 610041 China

**Keywords:** Ca^2+^ channel, Capacitance measurement, Casein kinase, Endocytosis, FM1-43 imaging, Syndapin/PACSIN

## Abstract

Endocytosis is controlled by a well-orchestrated molecular machinery, where the individual players as well as their precise interactions are not fully understood. We now show that syndapin I/PACSIN 1 is expressed in pancreatic β cells and that its knockdown abrogates β cell endocytosis leading to disturbed plasma membrane protein homeostasis, as exemplified by an elevated density of L-type Ca^2+^ channels. Intriguingly, inositol hexakisphosphate (InsP_6_) activates casein kinase 2 (CK2) that phosphorylates syndapin I/PACSIN 1, thereby promoting interactions between syndapin I/PACSIN 1 and neural Wiskott–Aldrich syndrome protein (N-WASP) and driving β cell endocytosis. Dominant-negative interference with endogenous syndapin I/PACSIN 1 protein complexes, by overexpression of the syndapin I/PACSIN 1 SH3 domain, decreases InsP_6_-stimulated endocytosis. InsP_6_ thus promotes syndapin I/PACSIN 1 priming by CK2-dependent phosphorylation, which endows the syndapin I/PACSIN 1 SH3 domain with the capability to interact with the endocytic machinery and thereby initiate endocytosis, as exemplified in β cells.

**Supplementary Information:**

The online version contains supplementary material available at 10.1007/s00018-022-04305-2.

## Introduction

Endocytosis is a fundamental cellular process. It serves to engulf extracellular substances such as nutrients, growth factors and pathogens. It also acts as a critical mechanism for density control of structural and functional components in the plasma membrane by exquisitely internalizing membrane proteins and lipids [1–3]. This is well exemplified by pancreatic β cells [4]. Mechanistically, endocytosis operates under direct control of a complex molecular network including the interaction of dynamin with PACSIN [2, 3, 5–7]. PACSIN is a cytoplasmic protein with a predicted molecular mass of 50 kilodaltons (kDa) [8, 9]. It was so named because it is a protein kinase C (PKC) and casein kinase 2 (CK2) substrate [8]. Originally, it was found that this protein is specifically enriched in synapses and physically associates with dynamin and therefore also termed syndapin [9]. Later, this neuron-specific isoform was called syndapin I/PACSIN 1 since other isoforms, syndapin II/PACSIN 2 and syndapin III/PACSIN 3, were detected in other tissues [5, 10–13]. However, the expression and function of syndapin I/PACSIN 1 in β cells are not known. Moreover, significant efforts in the field of β cell research have been devoted to the understanding of insulin exocytosis that is the primary and irreplaceable function of the β cell and inevitably coupled to endocytosis [4]. β Cell endocytosis is much less understood. The molecular details of the endocytic machinery in the β cell have not been well clarified.

Inositol hexakisphosphate (InsP_6_) is the fully monophosphorylated inositol species [14]. Intracellular InsP_6_ levels increase under stimulatory conditions and decrease in unstimulated cells [15–18]. InsP_6_ targets a number of specific binding proteins in the cell and acts as a player in multiple cellular processes including endocytosis, as exemplified in the pancreatic β cell [15, 18–25]. However, the mechanisms underlying InsP_6_-induced endocytosis are not known.

L-type Ca^2+^ channels are critical for electrically excitable and secretory β cells. These channels act as the pivot of a molecular network controlling the β cell primary function glucose-stimulated insulin secretion [15, 22, 26, 27]. They mediate Ca^2+^ influx in response to glucose stimulation to steer insulin-secretory granule trafficking to exocytotic sites and trigger its fusion with the β cell plasma membrane, the last event of glucose-stimulated insulin secretion [15, 22, 26, 27]. The β cell Ca_V_ channel-mediated Ca^2+^ influx induced by glucose stimulation is also involved in guaranteeing β cell maturity, growth and viability and ultimately adequate β cell mass and function by controlling β cell transcriptome, proteome, signalome and metabolome. β Cell L-type Ca^2+^ channels not only rely on their activity but also their density to fulfill their tasks. Their activity regulation has been thoroughly studied but their density control especially by endocytic trafficking is not clear.

The present work reports the following novel observations: (1) syndapin I/PACSIN 1 is present in β cells where it critically controls β cell endocytosis; (2) downregulation of syndapin I/PACSIN 1 leads to aberrant plasma membrane protein homeostasis, manifested as an elevated density of L-type Ca^2+^ channels; (3) InsP_6_ primes syndapin I/PACSIN 1 activation in endocytosis via CK2-dependent phosphorylation, which enables the syndapin I/PACSIN 1 SH3 domain to interact with endocytic players, exemplified by neural Wiskott–Aldrich syndrome protein (N-WASP).

## Materials and methods

### Pancreatic islet isolation

Mice and rats were anesthetized with CO_2_ and then killed by decapitation. Their abdomens were opened to expose the pancreas. Subsequently, in situ ductal perfusion was performed. Approximately, 5 and 15 ml collagenase solution (1.2 mg/ml; Roche, Basel, Switzerland) were injected into mouse and rat pancreas, respectively, through the common bile duct. The inflated pancreas was digested by shaking in collagenase solution for 20 min at 37 °C. Then, the digested pancreas was disintegrated by pipetting through a 5-ml pipette tip and rinsed with Hanks balanced salt solution (Invitrogen, Carlsbad, CA, USA) [28, 29]. The harvested islets were hand-picked and some of them were dispersed into single islet cells. Thereafter, both islets and dispersed islet cells were subjected to cultivation.

### Cultivation, transfection and electropermeabilization of islets and cells

HIT-T15, RINm5F, rodent islets and islet cells were cultivated in RPMI 1640 medium supplemented with 10% fetal bovine serum, 2 mM l-glutamine, and 100 U/100 µg/ml penicillin/streptomycin (Invitrogen). INS-1 cells were grown in RPMI 1640 medium containing the following additives: 10% fetal bovine serum, 2 mM l-glutamine, and 100 U/100 µg/ml penicillin/streptomycin, 10 mM HEPES, 1 mM sodium pyruvate and 50 μM β-mercaptoethanol (Invitrogen). MIN6-m9 cells were cultured in DMEM medium containing the following supplements: 10% fetal bovine serum, 100 U/100 µg/ml penicillin/streptomycin, 11 mM glucose and 0.0005% β-mercaptoethanol. The islets and cells were maintained at 37 °C in a humidified 5% CO_2_ incubator. HIT-T15, INS-1, MIN6-m9 and RINm5F cells were grown to approximately 70% confluence and then subjected to electropermeabilization, immunoprecipitation and immunoblot analysis.

Mouse islet β cells and RINm5F cells were plated on glass coverslips, cultivated for 24 h and then transfected with the plasmid encoding enhanced green fluorescence protein/wild-type syndapin I/PACSIN 1 SH3 domain (pEGFP/wtPCS1SH3) and the plasmid encoding enhanced green fluorescence protein/mutant syndapin I/PACSIN 1 SH3 domain (pEGFP/mtPCS1SH3), respectively. Wild-type syndapin I/PACSIN 1 SH3 cDNA encoding amino acids 376–441 and mutant syndapin I/PACSIN 1 SH3 cDNA encoding amino acids 376–441 with the P434L point mutation were generated by PCR amplification. The cDNAs were then cloned into pEGFP-C1 vector (Clontech, Palo Alto, CA, USA) to obtain pEGFP/wtPCS1SH3 and pEGFP/mtPCS1SH3. The cells cultured in RPMI 1640 medium were rinsed with transfection medium (Opti-MEM I, Invitrogen). pEGFP/wtPCS1SH3 and pEGFP/mtPCS1SH3 were transfected into cells in transfection medium using Lipofectamine LXT and PLUS reagent (Invitrogen). Cells were washed and refed with the RPMI 1640 medium after overnight transfection.

Two pairs of 21-mer siRNA duplexes targeting the rat syndapin I/PACSIN 1 (PACSIN siRNA #1, ID S132057 and syndapin I/PACSIN 1 siRNA #2, ID S132056) were designed and chemically synthesized by Applied Biosystems/Ambion (Austin, TX, USA). Their sequences were subjected to BLAST search to ensure their specificity. Silencer^®^ Select Negative Control siRNA (4390843), not targeting any gene product, and Silencer^®^ Select GAPDH Positive Control siRNA (4390849), efficiently silencing GAPDH in human, mouse, and rat cells, were purchased from Applied Biosystems/Ambion. RINm5F cells were reversely transfected with Lipofectamine^TM^ RNAiMAX. Briefly, negative control siRNA, syndapin I/PACSIN 1 siRNA #1 or syndapin I/PACSIN 1 siRNA #2 was mixed with Lipofectamine^TM^ RNAiMAX followed by 20-min incubation at room temperature. Subsequently, cells were added to the siRNA/LipofectamineTM RNAiMAX mixtures followed by gentle agitation and kept at 37 °C in a humidified 5% CO_2_ incubator. After 72 h, the transfected cells were grown to about 70% confluency and subjected to FM1-43 imaging and single-channel recording.

RINm5F cells were washed with an intracellular buffer (140 mM K-glutamate, 5 mM NaCl, 1 mM MgSO_2_, 25 mM HEPES, 2 mM Mg-ATP, 2 mM creatine phosphate and 10 U/ml creatine kinase). For intracellular exposure of cells to InsP_6_, cells were suspended in the intracellular buffer supplemented with 50 μM InsP_6_ and electroporated by five discharges of 1–3 kV at 2 μF. A 0.4% trypan blue solution (Sigma, St. Louis, Missouri, USA) was used to stain the electropermeabilized cells to ensure successful permeabilization. Subsequently, the electropermeabilized cells were subjected to live-cell confocal imaging of FM1-43.

### Immunocytochemistry and confocal microscopy

Double immunolabeling was performed on isolated rat and mouse islets as well as cultured rat and mouse islet cells. Isolated islets were fixed with 4% paraformaldehyde for 90 min. Cultured rat and mouse islet cells on glass coverslips were fixed in 2% paraformaldehyde for 30 min. Both the fixed islets and cultured cells were blocked with 5% normal goat serum (Sigma) for 1 h. The specimens were then double-labeled with rabbit polyclonal antibodies to syndapin I/PACSIN 1 (1:200) and guinea pig polyclonal antibodies to insulin (1:200; Dako, Glostrup, Denmark) at 4 °C overnight. A subsequent incubation of the specimens with goat anti-rabbit or anti-guinea pig IgG coupled to Alexa 488 or Alexa 633 (1:200; Molecular Probes, Eugene, OR, USA) proceeded for 20 min at room temperature. Omission of the primary antibodies or incubation with non-immune IgG from corresponding species was used as controls. Preabsorption of the anti-insulin (1:200) with bovine insulin (1000 µg/ml) was also performed to evaluate the specificity of the anti-insulin. The specimens were mounted in ProLong Gold Antifade (Molecular Probes) and visualized with a Leica TCS-SP5II-AOBS confocal laser-scanner connected to a Leica DM6000 CFS or a Leica TCS SP8 X confocal laser-scanner connected to a DMi8 microscope (Leica Microsystems Heidelberg GmbH, Mannheim, Germany). Alexa 488 and 633 linked to goat anti-rabbit and anti-guinea pig IgG were excited by a 488 and 633 nm laser line, respectively, and the resultant emissions were collected at 499–537 and 648–734 nm, respectively. Optical sections were captured using Leica HCX IRAPO L 25×/0.95 water, HCX PL APO 100×/1.44 oil and HC PL APO CS2 63×/1.3 GLYC objectives. The confocal images were processed and deconvoluted with Huygens Essential (Scientific Volume Imaging, Hilversum, The Netherlands).

Live-cell confocal imaging of FM1-43 was performed in RINm5F cells. Cells transfected with siRNAs underwent 30 min incubation with RPMI 1640 medium containing no glucose and then 10 min incubation with extracellular solution consisting of 135 mM NaCl, 3.6 mM KCl, 5 mM NaHCO_3_, 0.5 mM NaH_2_PO_4_, 0.5 mM MgCl_2_, 1.5 mM CaCl_2_, 10 HEPES and 0.1% bovine serum albumin. Subsequently, FM1-43 (Molecular Probes) was added at a concentration of 5 μM. Cells were stimulated for 5 min with either 10 mM glyceraldehyde plus 2.8 mM glucose or 30 mM KCl for endocytosis measurements. The whole procedure was conducted at 37 °C. The cells subjected to electropermeabilization and intracellular exposure to InsP_6_ were kept at about 4 °C. Then, these cells were exposed to 5 μM FM1-43 and endocytosis of the FM1-43-labeled plasma membrane of InsP_6_-containing cells was initiated by increasing the temperature of perifusion solution to 37 °C and measured with a Leica TCS-SP2 confocal laser-scanner connected to a Leica DMIRBE microscope or a Leica TCS SP8 X confocal laser-scanner connected to a DMi8 microscope (Leica). FM1-43 was excited by a 488 nm laser line and the resultant emission was captured using a Leica HC PL APO CS2 63×/1.3 GLYC objective at 540–650 nm [30]. FM1-43 imaging data were analyzed with FIJI/ImageJ. FM1-43 fluorescence in the intracellular compartment was segregated from that in the plasma membrane.

### Immunoprecipitation, SDS-PAGE and immunoblot analysis

The electropermeabilized RINm5F cells, following different treatments, were lysed in a lysis buffer (pH 7.5) consisting of 50 mM HEPES, 150 mM NaCl, 1 mM EGTA, 1 mM EDTA, 10% glycerol, 1% triton X-100, 1 mM PMSF and a protease inhibitor cocktail (Roche Diagnostics, Mannheim, Germany). The lysate was centrifuged at 800×*g* for 10 min at 4 °C to remove cell debris and nuclei. 1000 μg of lysate proteins were cleaned with 1 μg non-immune goat IgG (Santa Cruz Biotechnology, Santa Cruz, CA, USA) and 20 μl protein A/G PLUS-agarose beads (Santa Cruz Biotechnology). The cleaned samples were immunoprecipitated with goat polyclonal antibodies to syndapin I/PACSIN 1 (Santa Cruz Biotechnology) and non-immune goat IgG together with protein A/G PLUS-agarose beads. The resultant immunoprecipitates were subjected to SDS-PAGE/immunoblot analysis.

Adult male and female mice were killed by cervical dislocation. The pancreas, brain, heart, kidney, liver, lung, muscle and spleen were quickly dissected out. The pancreas was digested with collagenase (Boehringer Mannheim GmbH, Germany), and islets and exocrine tissue were hand-picked. The obtained tissues as well as insulin-secreting HIT-T15, INS-1, MIN6-m9 and RINm5F cells were homogenized on ice in 250 μl of a homogenization buffer (pH 7.4) consisting of 20 mM HEPES, 1 mM MgCl_2_, 2 mM EDTA, 250 mM sucrose, 1 mM PMSF and a protease inhibitor cocktail (Roche Diagnostics). Nuclei, unbroken cells, and debris in the homogenates were pelleted at 800×*g* for 10 min. The protein concentration of the resulting samples was determined with Bio-Rad protein assay reagent (Bio-Rad, Hercules, CA, USA). The samples were then denatured by heating at 96 °C for 3 min in SDS sample buffer and subjected to sodium dodecyl sulfate–polyacrylamide gel electrophoresis (SDS-PAGE) and immunoblot analysis. Briefly, 45–180 µg proteins were separated in discontinuous gels consisting of a 4% acrylamide stacking gel (pH 6.8) and a 8% acrylamide separating gel (pH 8.8). The separated proteins were then electroblotted to hydrophobic polyvinylidene difluoride membrane (Hybond-P; Amersham, Buckinghamshire, UK). The blots were blocked by incubation for 1 h with 5% non-fat milk powder in a washing buffer, containing 50 mM tris(hydroxymethyl)aminomethane, 150 mM NaCl and 0.05% Tween 20 (pH 7.5), and then incubated, overnight at 4 °C with affinity-purified rabbit polyclonal antibodies against syndapin I/PACSIN 1 (1:1000) [9] or rabbit monoclonal antibodies to N-WASP (1:1000, Cell Signaling Technology, Danvers, MA, USA). After washing, the blots were incubated with the secondary antibodies (horseradish peroxidase-conjugated goat anti-rabbit IgG; 1:50,000; Bio-Rad) at room temperature for 45 min. The immunoprecipitates with goat polyclonal antibodies to syndapin I/PACSIN 1 from RINm5F cells were also subjected to SDS-PAGE and immunoblot analysis with affinity-purified rabbit polyclonal antibodies against syndapin I/PACSIN 1 (1:1000), mouse monoclonal antibodies against phosphoserine (1:200, Qiagen, Valencia, CA, USA) or rabbit monoclonal antibodies to N-WASP. The immunoreactive bands were visualized with the ECL plus Western blotting detection system (Amersham).

### Electrophysiology

The conventional whole cell and cell-attached modes were employed for capacitance analysis and single-channel recording, respectively. Electrodes were made from borosilicate glass capillaries, fire-polished and coated with Sylgard close to their tips. The electrode resistance ranged between 4 and 6 MΩ when the pipettes were filled with the intracellular solutions. The electrode offset potential was corrected in extracellular solutions prior to gigaseal formation.

For capacitance analysis, mouse islet β cells, some pretreated with 25 μM TBB for 20 min and others transfected with pEGFP/wtPCS1SH3 and pEGFP/mtPCS1SH3 before capacitance recordings, were used. The cells expressing EGFP were selected for capacitance measurements. Whole-cell capacitance measurements were performed with an EPC-9 patch clamp amplifier together with LockIn extension of PULSE software (HEKA Elektronik, Lambrecht/Pfalz, Germany). The electrode solution was composed of (mM): 125 K-glutamate, 10 KCl, 10 NaCl, 1 MgCl_2_, 10 EGTA, 2 CaCl_2_, 3 Mg-ATP and 5 HEPES (pH 7.15). The resulting free Ca^2+^ concentration in the solution was 54 nM. Electrodes were filled with the electrode solution alone or together with 50 μM InsP_6_ (Sigma). The standard extracellular solution consisted of (in mM): 138 NaCl, 5.6 KCl, 1.2 MgCl_2_, 2.6 CaCl_2_, 5 HEPES and 5 glucose (pH 7.4). Cells were continuously perifused with the extracellular solution at a rate of 2 ml/min during the course of an experiment. The temperature of the extracellular solution was 34 °C, when measured in the position of recording electrodes. A sinewave stimulus (700 Hz, 25 mV peak-to-peak) was superimposed onto a DC holding potential of − 70 mV. The cell capacitance was recorded at low time resolution using X-chart plug-in module of PULSE software. Capacitance values acquired with LockIn during 0.2 s were averaged into an X-chart data point. The data were analyzed with a PC computer using IGOR Pro (WaveMetrics, Inc., Lake Oswego, OR, USA).

RINm5F cells transfected with syndapin I/PACSIN 1 siRNA or negative control siRNA were employed for single-channel measurements [31]. Electrodes were filled with a solution containing (in mM) 110 BaCl_2_, 10 TEA-Cl, and 5 HEPES (pH 7.4 with Ba(OH)_2_) as well as 10 µM Bay K8644. Single-channel recordings were performed with cells bathed in a depolarizing external recording solution, containing (in mM) 125 KCl, 30 KOH, 10 EGTA, 2 CaCl_2_, 1 MgCl_2_, and 5 HEPES–KOH (pH 7.15) as well as 10 µM Bay K8644. This solution was used to bring the intracellular potential to 0 mV. Single-channel currents were recorded with an Axopatch 200B amplifier (Molecular Devices, Foster City, CA, USA) at room temperature (about 22 °C). Acquisition and analysis of single-channel data were done using the software program pCLAMP 10 (Molecular Devices).

### Statistical analysis

All individual data and their mean ± SD were presented. Statistical significance was determined by one-way ANOVA, followed by least significant difference (LSD) test. When two groups were compared, unpaired Student’s *t* test or Mann–Whitney *U* test was employed. The significance level was set to 0.05.

## Results

### Syndapin I/PACSIN 1 is present in β cells

**Fig. 1:**
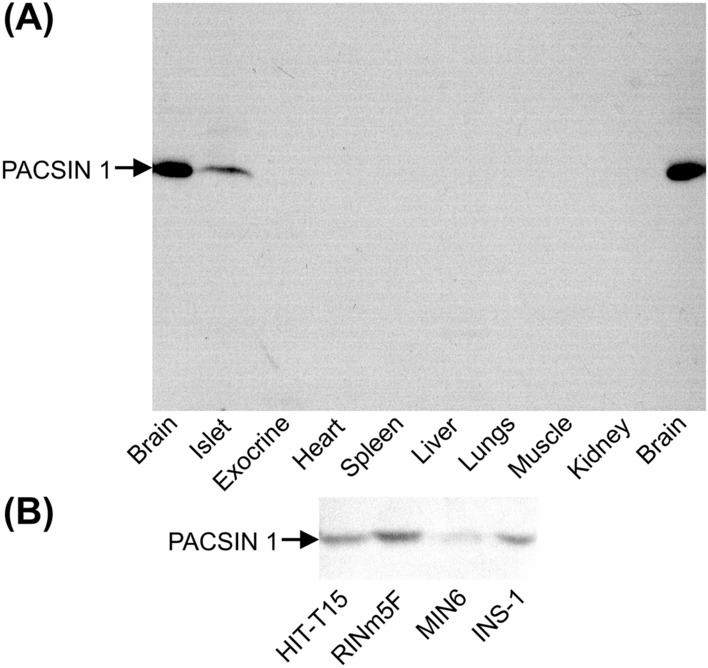
Immunoblot analysis of syndapin I/PACSIN 1 in pancreatic islets and insulin-secreting cells. **A** A representative immunoblot of syndapin I/PACSIN 1-immunoreactive bands in mouse pancreatic islets, brain, exocrine tissue, heart, spleen, liver, lung, muscle and kidney. **B** A sample immunoblot of syndapin I/PACSIN 1-immunoreactive bands in insulin-secreting HIT-T15, RINm5F, MIN6-m9 and INS-1 cells. The experiments were repeated three times

To examine if syndapin I/PACSIN 1 is expressed in β cells, we have performed immunoblot analysis and immunofluorescence labeling/confocal microscopy with syndapin I/PACSIN 1-specific antibodies. Immunoblot analysis of postnuclear homogenates from different mouse tissues revealed that anti-syndapin I/PACSIN 1 antibody recognized a protein band of about 50 kDa in both brain and pancreatic islets (Fig. 1A). However, this antibody detected no immunoreactive bands in exocrine pancreas, heart, kidney, liver, lung, muscle and spleen (Fig. 1A). It has been demonstrated that syndapin I/PACSIN 1 is specifically expressed in brain [8, 9]. The band visualized in islet samples exhibited a migration behavior identical to that of the band observed in brain extracts. This demonstrates that syndapin I/PACSIN 1 is also present in pancreatic islets. In addition to different mouse tissues, four commonly used insulin-secreting cell lines, HIT-T15, INS-1, MIN6-m9 and RINm5F, have also been subjected to immunoblot analysis. As shown in Fig. 1B, the anti-syndapin I/PACSIN 1 antibody revealed a protein band of about 50 kDa in all these cell lines. The strongest band was visualized in RINm5F cells and the weakest band was observed in MIN6-m9 cells (Fig. 1B).

Although the majority of islet cells are insulin-secreting β cells, the syndapin I/PACSIN 1 immunoreactivity revealed in islet cell homogenates by immunoblot analysis does not necessarily mean that β cells express this protein. Therefore, the localization of syndapin I/PACSIN 1 in both isolated pancreatic islets and cultured islet cells has been examined using immunofluorescence labeling/confocal microscopy/deconvolution analysis. Such techniques are employed not only to verify the presence of syndapin I/PACSIN 1 in islet β cells, but also to subcellularly localize syndapin I/PACSIN 1 in these cells.

**Fig. 2:**
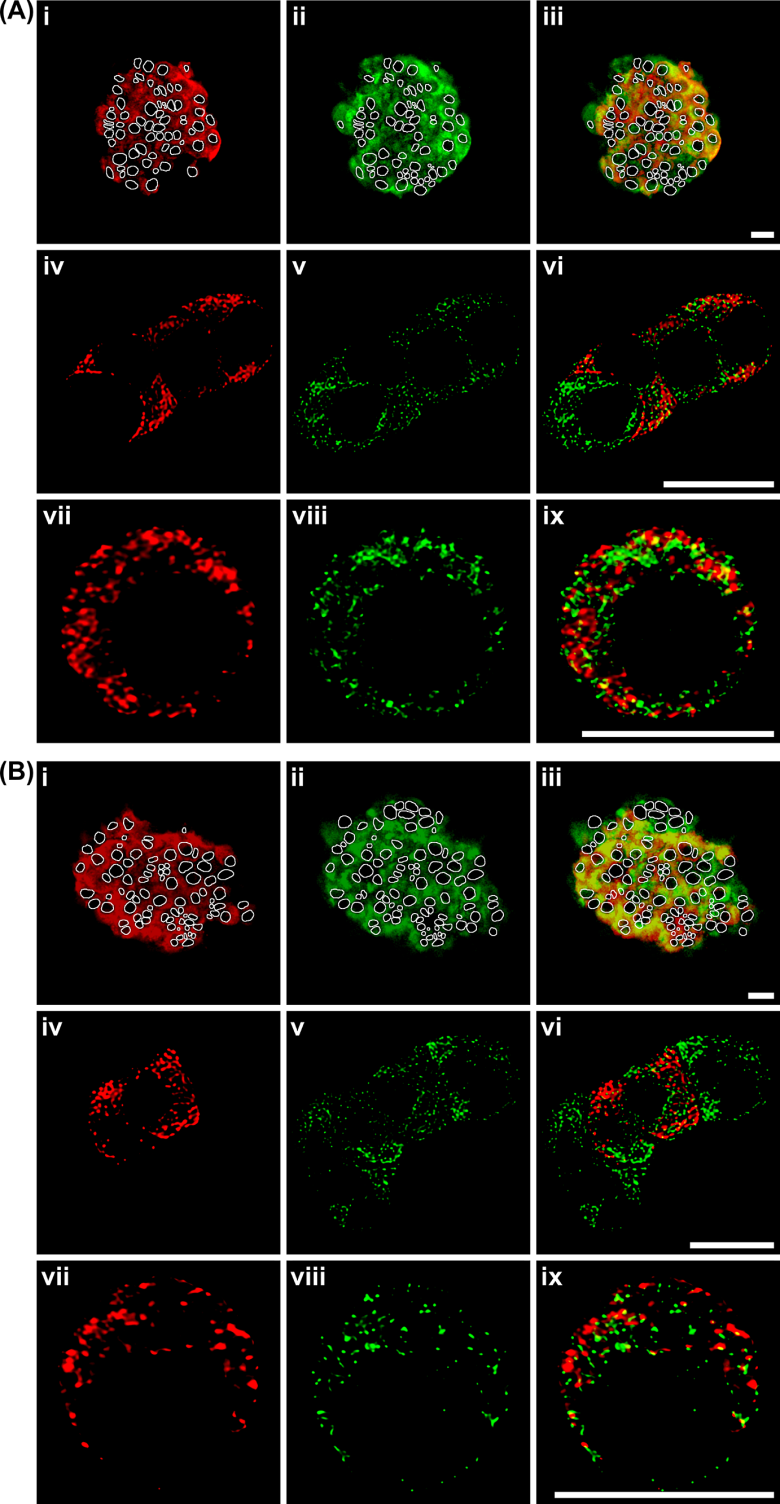
Immunocytochemical characterization of syndapin I/PACSIN 1 in mouse and rat islets and islet cells. **A** Distribution of insulin and syndapin I/PACSIN 1 immunofluorescence in isolated islets and cultured islet cells of mice. Confocal images of insulin (i), syndapin I/PACSIN 1 immunofluorescence (ii) and their overlay (iii) in isolated islets. Nucleus was marked with white lines. Deconvoluted confocal images of insulin (iv and vii), syndapin I/PACSIN 1 immunofluorescence (v and viii) and their overlay (vi and ix) in both cultured β and non-β cells. **B** Distribution of insulin and syndapin I/PACSIN 1 immunofluorescence in isolated islets and cultured islet cells of rats. Confocal images of insulin (i), syndapin I/PACSIN 1 immunofluorescence (ii) and their overlay (iii) in isolated islets. Nucleus was marked with white lines. Deconvoluted confocal images of insulin (iv and vii), syndapin I/PACSIN 1 immunofluorescence (v and viii) and their overlay (vi and ix) in both cultured β and non-β cells. Bars = 10 μm. The experiments were repeated four times

The specificity of guinea pig polyclonal antibodies against insulin and rabbit polyclonal antibodies against syndapin I/PACSIN 1 was verified in isolated islets and cultured islet cells incubated with the primary anti-insulin antibody preabsorbed with bovine insulin or the primary antibody-omitted solution followed by corresponding secondary antibodies. No specific staining was observed in cells incubated with the primary antibody preabsorbed with bovine insulin or the primary antibody-omitted solution (Supplementary Figs. 1 and 2). In experiments with islets isolated from both mouse (Fig. 2Ai–iii) and rat pancreases (Fig. 2Bi–iii), double staining with anti-insulin and anti-syndapin I/PACSIN 1 antibody revealed that most islet cells exhibited intense insulin immunofluorescence (Fig. 2Ai, Bi). However, all endocrine cells including β cells and other islet cells were labeled by the anti-syndapin I/PACSIN 1 antibody (Fig. 2Aii, Bii). The overlay of the insulin immunofluorescence and syndapin I/PACSIN 1 immunofluorescence image shows that the majority of cells were double-labeled, but some cells were only recognized by anti-syndapin I/PACSIN 1 antibody (Fig. 2Aiii, Biii). In characterization of cultured mouse and rat islet cells with a high-resolution approach, incubation with a mixture of anti-insulin and anti-syndapin I/PACSIN 1 antibody gave intense insulin immunofluorescence in some of the clustered cells (Fig. 2Aiv, Biv) and most of the single cells (Fig. 2Avii, Bvii) as well as intense syndapin I/PACSIN 1 immunofluorescence in all clustered cells (Fig. 2Av, Bv) and all single cells (Fig. 2Aviii, Bviii). Both insulin and syndapin I/PACSIN 1 immunofluorescence appeared in the cytoplasm with clear granule-like structures. A majority of syndapin I/PACSIN 1 immunofluorescence is separated from insulin immunofluorescence (Fig. 2Avi, Aix, Bvi, Bix). Only a small proportion of syndapin I/PACSIN 1 immunofluorescence colocalized with insulin immunofluorescence in the same subcellular structures (Fig. 2Avi, Aix, Bvi, Bix). Taken together, immunostaining data suggest that syndapin I/PACSIN 1 is expressed in all mouse and rat pancreatic islet cells including insulin-secreting β cells.

### Syndapin I/PACSIN 1 knockdown impairs intracellular FM1-43 accumulation in β cells

**Fig. 3:**
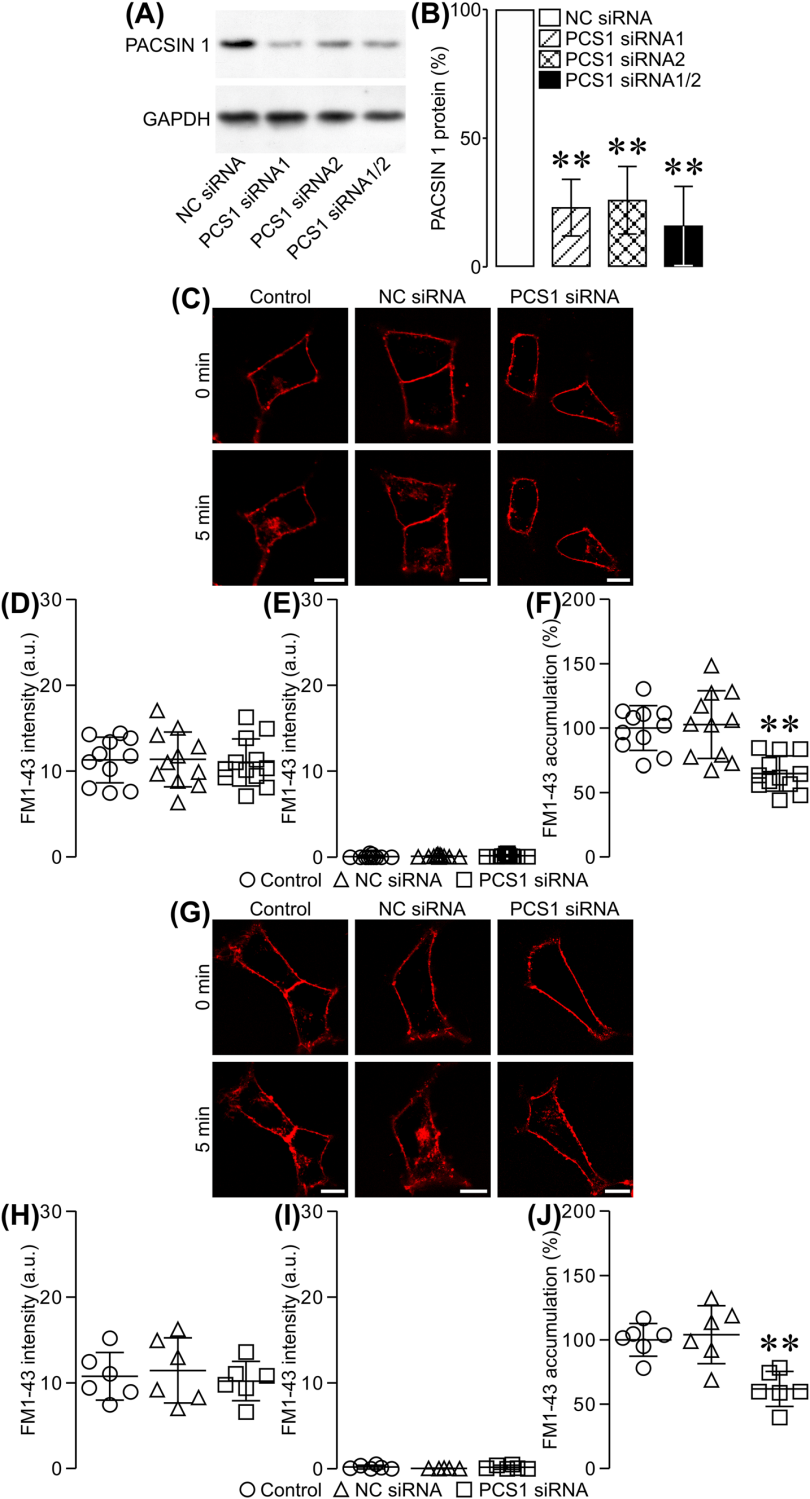
Effects of syndapin I/PACSIN 1 knockdown on intracellular FM1-43 accumulation in RINm5F cells. **A** Representative immunoblots of syndapin I/PACSIN 1- and GAPDH-immunoreactive bands in cells transfected with negative control siRNA (NC siRNA), syndapin I/PACSIN 1 siRNA #1 (PCS1 siRNA1), syndapin I/PACSIN 1 siRNA #2 (PCS1 siRNA2) and syndapin I/PACSIN 1 siRNA #1 plus syndapin I/PACSIN 1 siRNA #2 (PCS1 siRNA1/2). **B** Immunoblot quantifications of syndapin I/PACSIN 1 protein in NC siRNA- (*n* = 7), PCS1 siRNA1- (*n* = 7), PCS1 siRNA2- (*n* = 7), and PCS1 siRNA1/2-transfected cells (*n* = 7). **P < 0.01 versus NC siRNA. The experiments were repeated 3 times. **C** Sample confocal images of FM1-43 fluorescence acquired at 0 min and 5 min in a control, NC siRNA- and PCS1 siRNA-transfected cell after exposure to 10 mM D-glyceraldehyde and 2.8 mM glucose. **D** FM1-43 fluorescence intensity in the plasma membrane at 0 min after exposure to carbohydrates in control (*n* = 11), NC siRNA (*n* = 11) and PCS1 siRNA (*n* = 12) groups. **E** FM1-43 fluorescence intensity in the cytoplasm at 0 min after exposure to carbohydrates in control (*n* = 11), NC siRNA (*n* = 11) and PCS1 siRNA (*n* = 12) groups. **F** Quantification of intracellular FM1-43 accumulation for 5 min in control (*n* = 11), NC siRNA (*n* = 11) and PCS1 siRNA (*n* = 12) groups following stimulation with 10 mM glyceraldehyde and 2.8 mM glucose. **P < 0.01 versus control or NC siRNA group. The experiments were repeated 3 times. **G** Examples of confocal images of FM1-43 fluorescence registered at 0 min and 5 min in a control, NC siRNA- and PCS1 siRNA-transfected cell after stimulation with 30 mM KCl. **H** FM1-43 fluorescence intensity in the plasma membrane at 0 min after KCl stimulation in control (*n* = 6), NC siRNA (*n* = 6) and PCS1 siRNA (*n* = 6) groups. **I** FM1-43 fluorescence intensity in the cytoplasm at 0 min after KCl stimulation in control (*n* = 6), NC siRNA (*n* = 6) and PCS1 siRNA (*n* = 6) groups. **J** Quantification of intracellular FM1-43 accumulation for 5 min in control (*n* = 6), NC siRNA (*n* = 6) and PCS1 siRNA (*n* = 6) groups following stimulation with 30 mM KCl. **P < 0.01 versus control group or NC siRNA group. Bar = 10 μm. The experiments were repeated 3 times

The presence of syndapin I/PACSIN 1 in β cells immediately raises the question whether it regulates β cell endocytosis. To answer this question, we downregulated syndapin I/PACSIN 1 expression by applying RNA interference-mediated gene silencing. Figure 3A, B shows that transfection with two syndapin I/PACSIN 1 siRNAs (PCS1 siRNAs) significantly decreased syndapin I/PACSIN 1 expression at the protein level as compared to that with negative control siRNA (NC siRNA) in insulin-secreting RINm5F cells.

Following the satisfactory knockdown of syndapin I/PACSIN 1, we performed live-cell confocal imaging of the endocytic marker FM1-43 to characterize endocytic processes in cells. β Cell endocytosis was evoked by extracellular application of 10 mM glyceraldehyde together with 2.8 mM glucose. As shown in Fig. 3C–E, FM1-43 intensively labeled the plasma membrane of control cells and those transfected with NC siRNA or PCS1 siRNA, but negligibly entered the cytoplasm before addition of carbohydrates (0 min). FM1-43 fluorescence intensity in either the plasma membrane or the cytoplasm did not significantly differ between different groups (Fig. 3D, E). Importantly, FM1-43 was substantially internalized into cells in all three groups, but to different degrees, at 5 min after carbohydrate stimulation (Fig. 3C, F). There was a significantly reduced internalization of FM1-43 into cells transfected with PCS1 siRNA compared to non-transfected or NC siRNA-transfected cells (Fig. 3C, F). Hence, downregulation of syndapin I/PACSIN 1 expression dramatically dampens β cell endocytosis induced by a high carbohydrate challenge. β Cell endocytosis was also provoked by stimulation with 30 mM KCl. Figure 3G–I shows that the plasma membrane of cells subjected to different treatments was strongly labeled with FM1-43, but the cytoplasm was not prior to depolarization with KCl (0 min). However, intracellular FM1-43 accumulation in PCS1 siRNA-transfected cells was significantly decreased compared to that in non-transfected cells or cells transfected with NC siRNA (Fig. 3G, J). This clearly shows that a decrease in syndapin I/PACSIN 1 markedly reduces β cell endocytosis following K^+^ depolarization.

### Syndapin I/PACSIN 1 knockdown results in an elevated density of L-type Ca^2+^ channels in the β cell plasma membrane

**Fig. 4:**
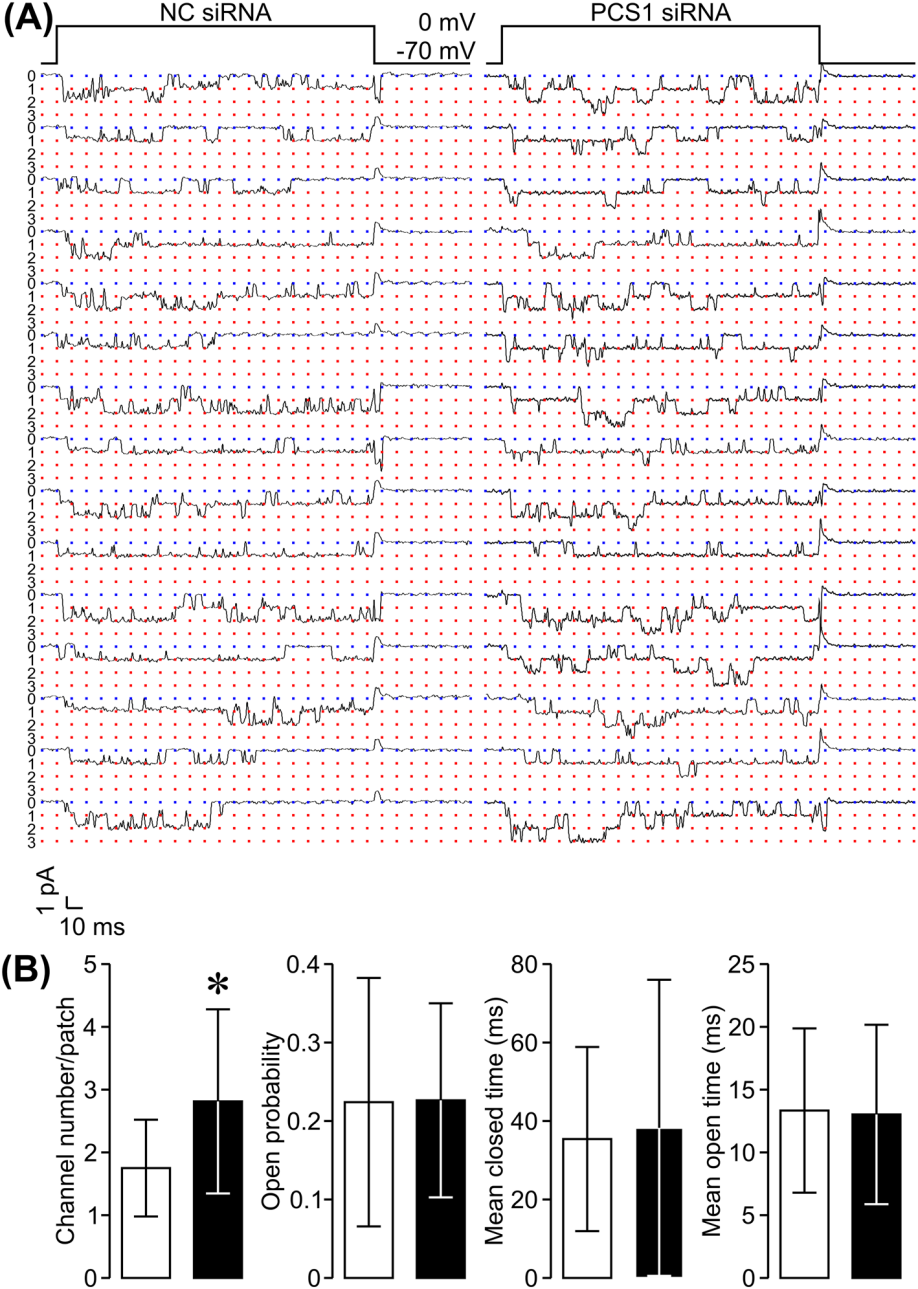
Effects of syndapin I/PACSIN 1 knockdown on density of L-type Ca^2+^ channels in β cell plasma membrane. **A** Examples of unitary L-type Ca^2+^ channel currents detected in plasma membrane patches attached to either a negative control siRNA- (NC siRNA-) transfected cell or a cell transfected with syndapin I/PACSIN 1 siRNA in the presence of the selective L-type Ca^2+^ channel activator Bay K8644 at 10 µM. **B** Average number, open probability, mean closed time and mean open time of unitary L-type Ca^2+^ channels measured in plasma membrane patches of cells following transfection with either NC siRNA (*n* = 16) or syndapin I/PACSIN 1 siRNA (*n* = 16) in the presence of the selective L-type Ca^2+^ channel activator Bay K8644 at 10 µM. *P < 0.05 versus NC siRNA. The experiments were repeated 5 times

The question now arises as to what is the physiological importance of syndapin I/PACSIN 1-mediated endocytosis in β cells. We tackled this question by evaluating the effect of the syndapin I/PACSIN 1 knockdown-caused impairment of β cell endocytosis on the density of L-type Ca^2+^ channels in the β cell plasma membrane. We analyzed unitary L-type Ca^2+^ channel currents, characterized by a large unitary Ba^2+^ conductance with long-lasting openings, in cells transfected with syndapin I/PACSIN 1 siRNA and in negative control siRNA-transfected cells following 1 h incubation with 10 mM glyceraldehyde plus 2.8 mM glucose. To reliably estimate the density of L-type Ca^2+^ channels in the β cell plasma membrane, 10 µM Bay K8644, a selective L-type Ca^2+^ channel activator, was included in both the electrode solution and the extracellular solution to maximally activate L-type Ca^2+^ channels in the recorded plasma membrane patches. We observed more L-type Ca^2+^ channels, reflected by more layers of unitary Ba^2+^ currents, in plasma membrane patches of syndapin I/PACSIN 1 siRNA-transfected cells than in those of cells transfected with negative control siRNA (Fig. 4A). The average number of unitary L-type Ca^2+^ channels in plasma membrane patches of syndapin I/PACSIN 1 siRNA-transfected cells was significantly greater than those of cells transfected with negative control siRNA (Fig. 4B). There was no significant difference in the open probability, mean open time and mean closed time of unitary L-type Ca^2+^ channels between cells transfected with syndapin I/PACSIN 1 siRNA and negative control siRNA-transfected cells (Fig. 4B). These data thus suggest that the syndapin I/PACSIN 1 knockdown-induced impairment in β cell endocytosis leads to disturbed plasma membrane protein homeostasis, as exemplified by the elevated density of β cell L-type Ca^2+^ channels.

### CK2 inhibition attenuates InsP_6_-induced phosphorylation of syndapin I/PACSIN 1, interaction of syndapin I/PACSIN 1 with N-WASP, reduction of cell membrane capacitance and intracellular FM1-43 accumulation in β cells

**Fig. 5:**
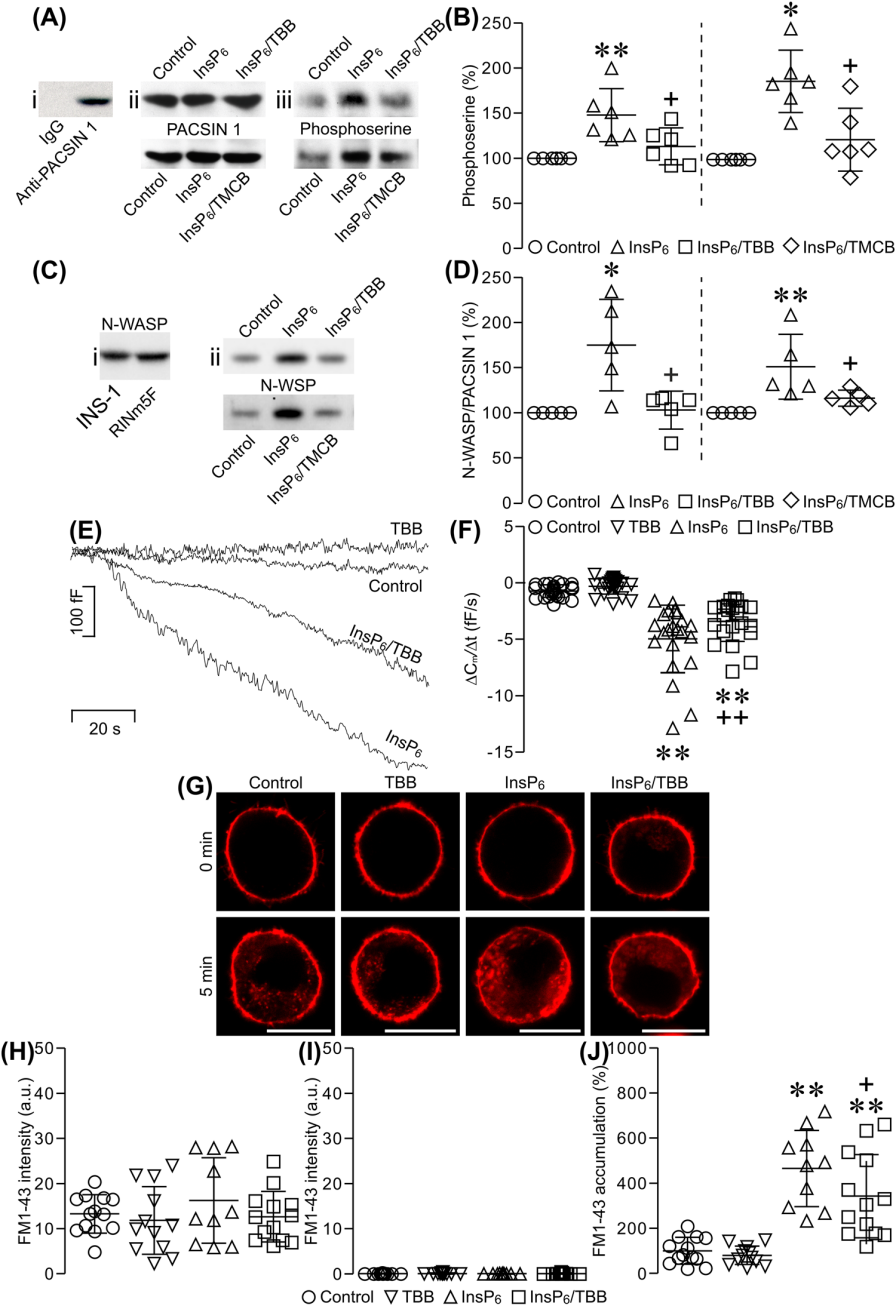
Effects of inhibition of CK2 on InsP_6_-induced phosphorylation of syndapin I/PACSIN 1, interaction of syndapin I/PACSIN 1 with N-WASP, reduction of β cell capacitance and intracellular FM1-43 accumulation. **A** A representative immunoblot of syndapin I/PACSIN 1-immunoreactive bands (i) in immunoprecipitates with non-immune goat IgG or goat polyclonal anti-syndapin I/PACSIN 1 antibody from RINm5F cells. Sample immunoblots of syndapin I/PACSIN 1- (ii) and phosphoserine-immunoreactive bands (iii) in immunoprecipitates with goat polyclonal anti-syndapin I/PACSIN 1 antibody from electropermeabilized RINm5F cells treated with a vehicle solution (control), 50 μM InsP_6_, 50 μM InsP_6_/25 μM TBB or 50 μM InsP_6_/10 μM TMCB. **B** Immunoblot quantifications of phosphoserine immunoreactivity in vehicle control (*n* = 6), 50 μM InsP_6_ (*n* = 6), 50 μM InsP_6_/25 μM TBB (*n* = 6) and 50 μM InsP_6_/10 μM TMCB (*n* = 6) groups. *P < 0.05 and **P < 0.01 versus control, ^+^*P* < 0.05 versus 50 μM InsP_6_. The experiments were repeated 3 times. **C** A representative immunoblot of N-WASP-immunoreactive bands in RINm5F and INS-1 cells (i). Sample immunoblots of N-WASP-immunoreactive bands in immunoprecipitates with goat polyclonal anti-syndapin I/PACSIN 1 antibody from electropermeabilized RINm5F cells treated with a vehicle solution (control), 50 μM InsP_6_ alone, 50 μM InsP_6_/25 μM TBB or 50 μM InsP_6_/10 μM TMCB (ii). **D** Immunoblot quantification of N-WASP immunoreactivity in vehicle control (*n* = 5), 50 μM InsP_6_ (*n* = 5), 50 μM InsP_6_/25 μM TBB (*n* = 5) or 50 μM InsP_6_/10 μM TMCB (*n* = 5) groups. *P < 0.05 and **P < 0.01 versus control. ^+^*P* < 0.05 versus 50 μM InsP_6_. The experiments were repeated 3 times. **E** Sample capacitance traces registered in a cell pretreated with 25 µM TBB, a control cell, a 25 μM TBB-pretreated cell subjected to 50 μM InsP_6_ (50 μM InsP_6_/25 μM TBB) and a cell treated with 50 μM InsP_6_. **F** Summary graph of the capacitance reduction rate in control (*n* = 25), 25 μM TBB (*n* = 24), 50 μM InsP_6_ (*n* = 22) and 50 μM InsP_6_/25 μM TBB (*n* = 22) groups. **P < 0.01 versus control or 25 µM TBB, ^++^*P* < 0.01 versus 50 µM InsP_6_. The experiments were repeated 12 times. **G** Sample confocal images of FM1-43 fluorescence acquired at 0 min and 5 min after initiation of endocytosis in control, 50 μM InsP_6_-, 25 μM TBB- or 50 μM InsP_6_/25 μM TBB-treated cell. **H** FM1-43 fluorescence intensity in the plasma membrane at 0 min after initiation of endocytosis in control (*n* = 13), 25 μM TBB (*n* = 12), 50 µM InsP_6_ (*n* = 10) and 50 µM InsP_6_/TBB (*n* = 13) groups. **I** FM1-43 fluorescence intensity in the cytoplasm at 0 min after initiation of endocytosis in control (*n* = 13), 25 μM TBB (*n* = 12), 50 µM InsP_6_ (*n* = 10) and 50 µM InsP_6_/TBB (*n* = 13) groups. **J** Quantification of intracellular FM1-43 accumulation for 5 min after initiation of endocytosis in control (*n* = 13), 50 μM InsP_6_ (*n* = 12), 25 μM TBB (*n* = 10) and 50 μM InsP_6_/25 μM TBB (*n* = 13) groups. **P < 0.01 versus control or 25 µM TBB, ^+^*P* < 0.05 versus 50 µM InsP_6_. Bar = 10 μm. The experiments were repeated 4 times

To mechanistically dissect how syndapin I/PACSIN 1 mediates endocytosis, we have explored if syndapin I/PACSIN 1 undergoes phosphorylation and consequently gains competence in interactions with other endocytic players in InsP_6_-induced endocytosis, which is the major type of β cell endocytosis. We immunoprecipitated syndapin I/PACSIN 1 from RINm5F cells and quantified phosphorylation of this protein with phospho-specific antibodies. As shown in Fig. 5Ai, polyclonal anti-syndapin I/PACSIN 1 antibody immunoprecipitated abundant syndapin I/PACSIN 1. In contrast, non-immune IgG could not pull down detectable quantities of this protein (Fig. 5Ai). Such selective immunoprecipitation of syndapin I/PACSIN 1 allowed us to examine InsP_6_-induced syndapin I/PACSIN 1 phosphorylation by CK2 in RINm5F cells with phospho-specific antibodies in combination with the CK2 inhibitors 4,5,6,7-tetrabromobenzotriazole (TBB) and TMCB. Figure 5Aii shows that polyclonal anti-syndapin I/PACSIN 1 antibody immunoprecipitated equal amounts of syndapin I/PACSIN 1 from electropermeabilized RINm5F cells treated with 50 μM InsP_6_, 50 μM InsP_6_ plus 25 μM TBB, 50 μM InsP_6_ plus 10 μM TMCB or a vehicle solution. Figure 5Aiii illustrates that mouse monoclonal antibodies against phosphoserine recognized syndapin I/PACSIN 1 immunoprecipitated from electropermeabilized RINm5F cells subjected to the four different treatments. Syndapin I/PACSIN 1 immunoprecipitated from cells treated with InsP_6_ displayed a significant increase in phosphoserine immunoreactivity in comparison with that from control cells (Fig. 5Aiii, B). The intensity of phosphoserine immunoreactivity in syndapin I/PACSIN 1 immunoprecipitated from cells exposed to InsP_6_ plus either TBB or TMCB was significantly lower than that in InsP_6_-treated cells (Fig. 5Aiii, B). These data verify that β cell syndapin I/PACSIN 1 is a suitable substrate for CK2 whose activity is stimulated by InsP_6_.

Subsequently, we evaluated if phosphorylated syndapin I/PACSIN 1 increases its competence in interactions with the important endocytic player N-WASP using immunoprecipitation and immunoblot analysis (Fig. 5C, D). Immunoblot analysis detected a clear N-WASP-immunoreactive band in RINm5F and INS-1 cells (Fig. 5Ci). Importantly, N-WASP co-immunoprecipitated with polyclonal anti-syndapin I/PACSIN 1 antibody from electropermeabilized RINm5F cells treated with 50 μM InsP_6_ significantly increased in comparison to that from control cells (Fig. 5Cii, D). Furthermore, this increase was effectively abolished by 25 μM TBB or 10 μM TMCB (Fig. 5Cii, D). These data reveal that phosphorylated syndapin I/PACSIN 1 interacts with N-WASP more strongly.

InsP_6_-induced syndapin I/PACSIN 1 phosphorylation by CK2 and consequent interaction with N-WASP promoted us to investigate if such phosphorylation mediates InsP_6_-induced endocytosis in β cells. We have previously demonstrated that intracellular application of InsP_6_ dose-dependently induces dynamin I-mediated endocytosis, as visualized by capacitance analysis in mouse islet β cells [23]. 100 μM InsP_6_ gave maximal endocytosis when the cytoplasmic free Ca^2+^ concentration ([Ca^2+^]_i_) was clamped at 54 nM [23]. In the present work, a sub-peak dose of 50 μM InsP_6_ was applied to evoke endocytosis in β cells where [Ca^2+^]_i_ was also clamped to 54 nM. Under such experimental conditions, InsP_6_-induced endocytosis was evaluated by capacitance analysis in β cells in the absence or presence of the CK2 inhibitor TBB. Figure 5E illustrates that a control cell or a cell pretreated with 25 μM TBB displayed a slight reduction in cell capacitance, registered with a pipette filled with a standard internal solution without InsP_6_. In contrast, a cell treated with 50 μM InsP_6_ exhibited a decrease in cell capacitance (Fig. 5E). This reflects that intracellular application of InsP_6_-induced endocytosis indicated by a time-dependent reduction in plasma membrane area mirrored by a gradual decrease in cell capacitance. Interestingly, a 25 μM TBB-pretreated cell exposed to 50 μM InsP_6_ showed less drop in cell capacitance than a cell treated with InsP_6_ alone (Fig. 5E). As illustrated in Fig. 5F, the capacitance reduction rate is small in either the control group or the group pretreated with 25 μM TBB in the absence of InsP_6_. In contrast, a significantly faster reduction in cell capacitance occurred in the 50 μM InsP_6_-treated group with or without 25 μM TBB pretreatment, in comparison with the control group or the group pretreated with TBB in the absence of InsP_6_. These data are well in accordance with our previous finding that InsP_6_, ranging from 20 to 200 μM, dose-dependently evokes capacitance reduction in mouse islet β cells [23]. Importantly, 50 μM InsP_6_ induced a significantly slower reduction in cell capacitance in the group pretreated with TBB in comparison with that in the group without TBB pretreatment (Fig. 5F). To substantiate the above data, cellular uptake of FM1-43 was monitored using live-cell confocal imaging. Control cells and those treated with 25 μM TBB, 50 μM InsP_6_ or 50 μM InsP_6_ plus 25 μM TBB displayed similarly intense FM1-43 labeling in their plasma membranes and very little, if any FM1-43, cytoplasmic labeling before initiation of endocytosis (0 min) (Fig. 5G–I). By contrast, at 5 min after initiation of endocytosis, cells exposed to 50 μM InsP_6_ intracellularly took up a significantly greater amount of FM1-43 in comparison to control and TTB-treated cells (Fig. 5G, J). Importantly, the InsP_6_-induced uptake of FM1-43 was significantly counteracted by co-application of TBB (Fig. 5G, J). Altogether, these data corroborate that InsP_6_ evokes β cell endocytosis, at least in part, by activation of CK2.

### Recombinant syndapin I/PACSIN 1 SH3 domain slows down InsP_6_-induced capacitance reduction and intracellular FM1-43 accumulation in β cells

Proline-rich motifs of dynamin interact with SH3 domains of other endocytic proteins to operate dynamin-mediated endocytosis [3, 32]. This made us question if the syndapin I/PACSIN 1 SH3 domain acts as an important player in dynamin-mediated β cell endocytosis. We answered this question by combining genetic transformation of β cells with pEGFP/wtPCS1SH3 or pEGFP/mtPCS1SH3, cell capacitance measurements and live-cell confocal imaging of FM1-43 uptake. To elicit dynamin-mediated endocytosis, 50 μM InsP_6_, under conditions where [Ca^2+^]_i_ was clamped to 54 nM, was introduced into mock-treated β cells and those expressing wtPCS1SH3 or mtPCS1SH3.

**Fig. 6:**
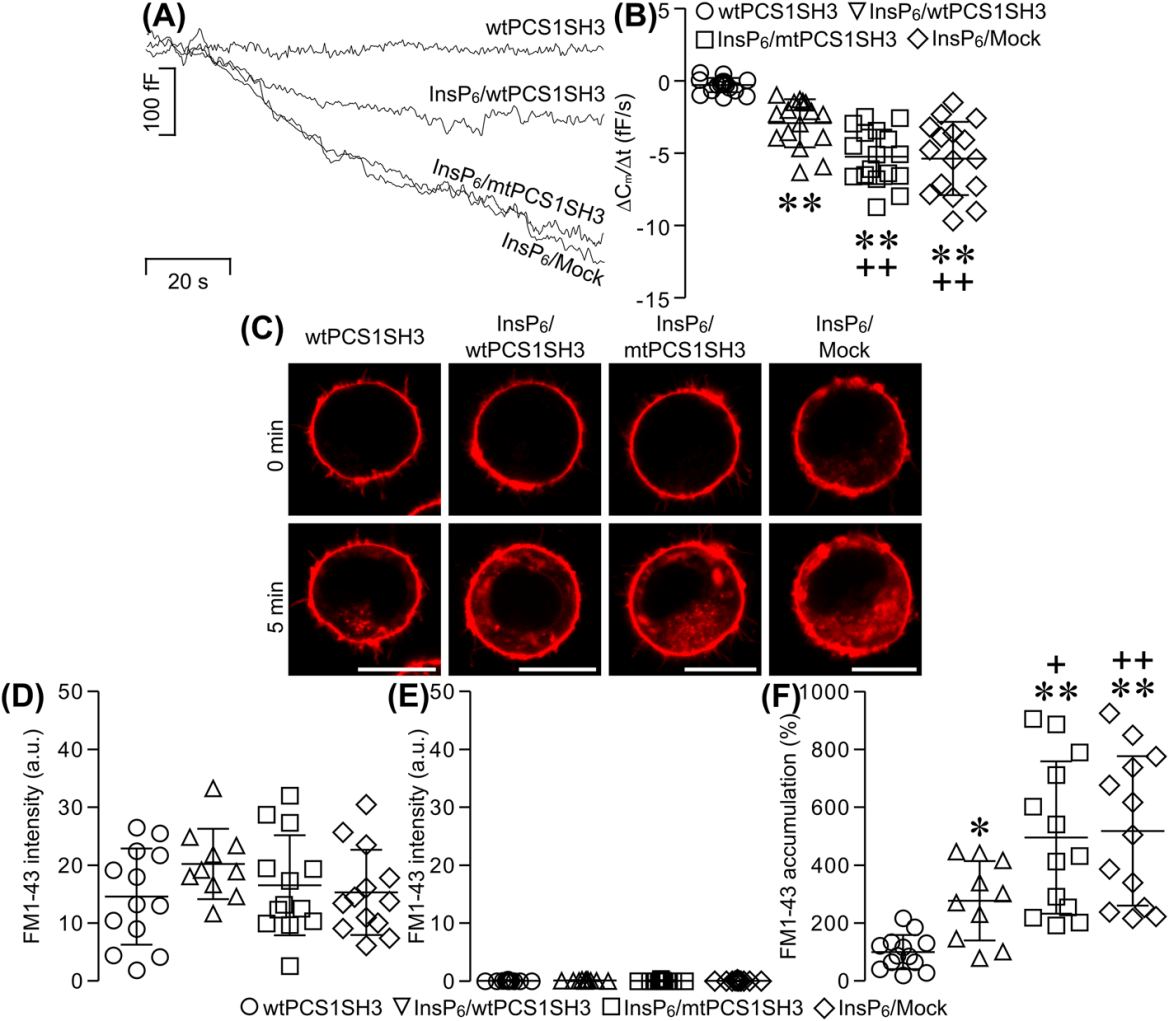
Effects of expression of syndapin I/PACSIN 1 SH3 domain on InsP_6_-induced reduction of β cell capacitance and intracellular FM1-43 accumulation. **A** Sample capacitance traces obtained in a pEGFP/wtPCS1SH3-transfected cell in the absence of InsP_6_ (wtPCS1SH3), a cell transfected with pEGFP/wtPCS1SH3 following intracellular application of 50 μM (50 µM InsP_6_/wtPCS1SH3), a pEGFP/mtPCS1SH3-transfected cell intracellularly exposed to 50 μM InsP_6_ (50 µM InsP_6_/mtPCS1SH3) and a mock-transfected cell intracellularly exposed to 50 μM InsP_6_ (50 µM InsP_6_/Mock). **B** Summary graph of the capacitance reduction rate in wtPCS1SH3 (*n* = 17), 50 µM InsP_6_/wtPCS1SH3 (*n* = 16), 50 µM InsP_6_/mtPCS1SH3 (*n* = 17) and 50 µM InsP_6_/Mock (*n* = 16) groups. **P < 0.01 versus wtPCS1SH3, ^++^*P* < 0.01 versus 50 µM InsP_6_/wtPCS1SH3. The experiments were repeated 8 times. **C** Sample confocal images of FM1-43 fluorescence acquired at 0 min and 5 min after initiation of endocytosis in wtPCS1SH3-, 50 µM InsP_6_/wtPCS1SH3-, 50 µM InsP_6_/mtPCS1SH3- or 50 µM InsP_6_/Mock-treated cell. **D** FM1-43 fluorescence intensity in the plasma membrane at 0 min after initiation of endocytosis in wtPCS1SH3 (*n* = 13), 50 µM InsP_6_/wtPCS1SH3 (*n* = 10), 50 µM InsP_6_/mtPCS1SH3 (*n* = 13) and 50 µM InsP_6_/Mock (*n* = 13) groups. **E** FM1-43 fluorescence intensity in the cytoplasm at 0 min after initiation of endocytosis in wtPCS1SH3 (*n* = 13), 50 µM InsP_6_/wtPCS1SH3 (*n* = 10), 50 µM InsP_6_/mtPCS1SH3 (*n* = 13) and 50 µM InsP_6_/Mock (*n* = 13) groups. **F** Quantification of intracellular FM1-43 accumulation for 5 min after initiation of endocytosis in wtPCS1SH3 (*n* = 13), 50 µM InsP_6_/wtPCS1SH3 (*n* = 10), 50 µM InsP_6_/mtPCS1SH3 (*n* = 13) and 50 µM InsP_6_/Mock (*n* = 13) groups. *P < 0.05 and **P < 0.01 versus wtPCS1SH3, ^+^*P* < 0.05 and ^++^*P* < 0.01 versus 50 µM InsP_6_/wtPCS1SH3. Bar = 10 μm. The experiments were repeated 4 times

As shown in Fig. 6A, a pEGFP/wtPCS1SH3-transfected cell did not show any appreciable changes in cell capacitance, which was recorded with a pipette containing a standard internal solution without InsP_6_. In contrast, either a mock-transfected cell or a cell transfected with pEGFP/mtPCS1SH3 displayed a gradual decrease in cell capacitance with similar time constants and amplitudes following intracellular application of 50 μM InsP_6_. Furthermore, a cell expressing wtPCS1SH3 also exhibited an appreciable decrease in cell capacitance in the presence of 50 μM InsP_6_. However, this response was markedly weaker than that evoked by the same concentration of InsP_6_ in either the mock-transfected cell or the pEGFP/mtPCS1SH3-transfected cell. Figure 6B summarizes the reduction rate of cell capacitance under the different experimental conditions. The capacitance reduction rate is negligible in cells expressing wtPCS1SH3 in the absence of InsP_6_. However, 50 μM InsP_6_ evoked a significantly faster reduction in cell capacitance in the other three groups including the pEGFP/wtPCS1SH3-, the pEGFP/mtPCS1SH3- and the mock-transfection group. Interestingly, the InsP_6_-induced capacitance reduction in the pEGFP/wtPCS1SH3-transfection group is significantly slower than that in the pEGFP/mtPCS1SH3- and the mock-transfection group. There is no significant difference in the capacitance reduction rate between the pEGFP/mtPCS1SH3- and the mock-transfection group. As shown in Fig. 6C–E, cells transfected with either pEGFP/wtPCS1SH3 or pEGFP/mtPCS1SH3 and mock-treated cells in the presence or absence of 50 μM InsP_6_ exhibited more or less the same level of FM1-43 fluorescence in their plasma membranes, but weak FM1-43 fluorescence in their intracellular compartment before endocytosis occurred (0 min). Furthermore, at 5 min after initiation of endocytosis, intracellular exposure to 50 μM InsP_6_ induced significantly more FM1-43 internalization in pEGFP/wtPCS1SH3-, pEGFP/mtPCS1SH3- and mock-transfected cells (Fig. 6C, F). Importantly, the InsP_6_-induced internalization of FM1-43 was significantly reduced by expression of pEGFP/wtPCS1SH3 (Fig. 6C, F). Taken together, our data demonstrate that exogenously expressed wtPCS1SH3, which strongly binds proline-rich motifs of endocytic proteins and ablates syndapin I/PACSIN 1-mediated endocytosis [5, 9], effectively interferes with InsP_6_-induced β cell endocytosis. The specificity of this dominant-negative interference is verified by the fact that single amino acid substitution mutant (P434L) of the syndapin I/PACSIN 1 SH3 domain, which is then incapable of interacting with proline-rich motifs of endocytic proteins [5, 9], is unable to influence InsP_6_-induced β cell endocytosis.

## Discussion

**Fig. 7:**
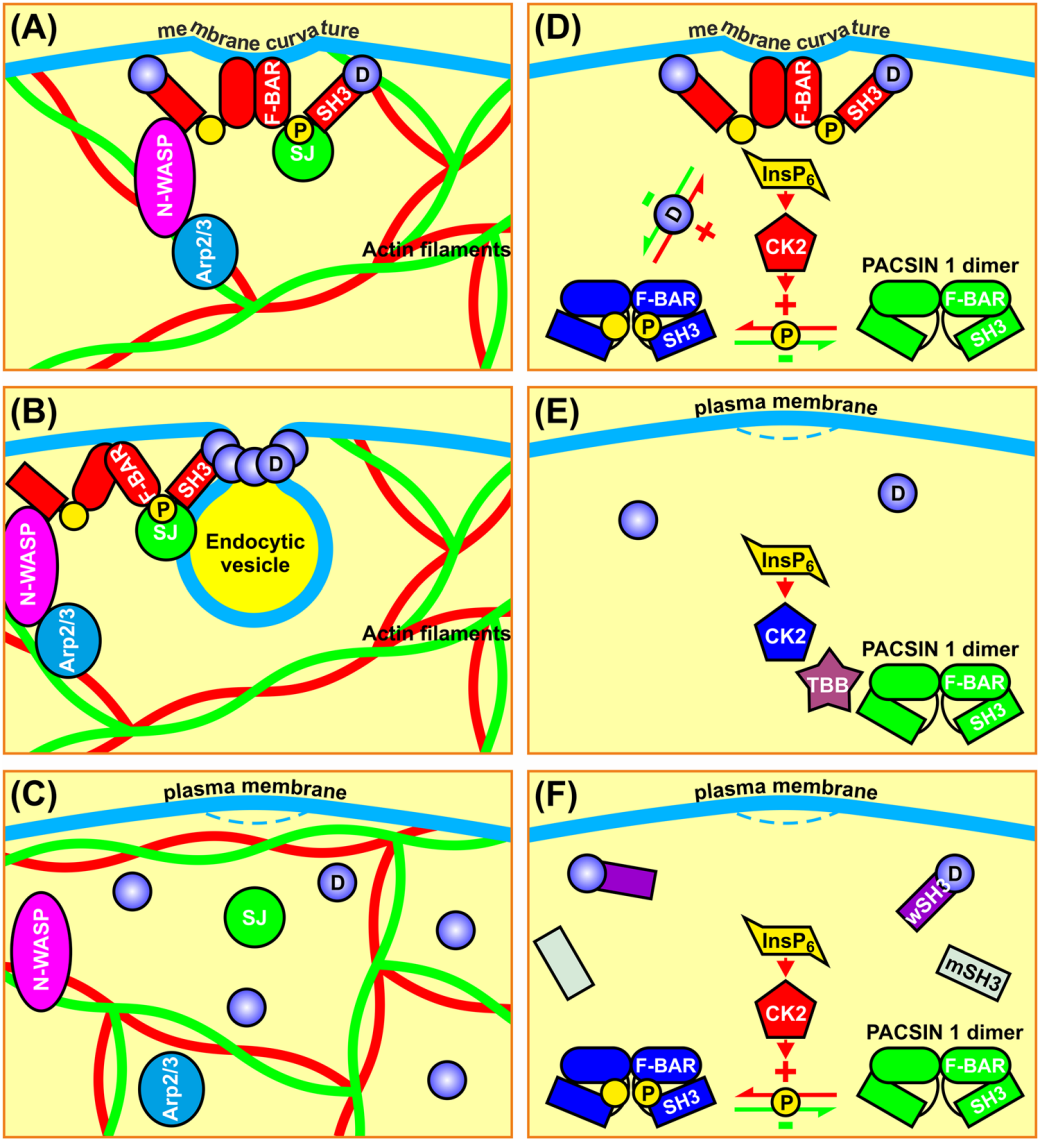
Schematic diagram illustrating the mechanisms of syndapin I/PACSIN 1-mediated β cell endocytosis. **A**, **B** The interaction of syndapin I/PACSIN 1 SH3 domain (red rectangle) with dynamin (blue circle) induces and stabilizes membrane curvature via dimerized syndapin I/PACSIN 1 F-BAR domain (red ellipse) (**A**). This interaction powers up dynamin to pinch off endocytic vesicles. In addition, the syndapin I/PACSIN 1 SH3 domains are physically associated with the Arp2/3 complex activator N-WASP to release autoinhibition of Arp2/3 complex [34] and thereby rearranging actin filaments [12, 35–37]. This process spatially organizes the endocytic machinery, builds up structural support for membrane topologies, removes local barriers to endocytic vesicle formation, and creates the force and directionality for vesicle fission and liberation (**B**) [12, 35–37]. **C** Syndapin I/PACSIN 1 knockdown significantly abrogates β cell endocytosis. **D** Syndapin I/PACSIN 1 homodimerizes via the intermolecular connection between two F-BAR domains (green ellipse) and adopts a closed conformation by the intramolecular binding of the SH3 domain (green rectangle) to the F-BAR domain without endocytic activity [38, 39]. Homodimeric syndapin I/PACSIN 1 opening requires not only binding of the syndapin I/PACSIN 1 SH3 domain to the proline-rich motif of dynamin I [38, 39] but also phosphorylation (yellow circle) of syndapin I/PACSIN 1 by InsP_6_-activated CK2. The SH3 domain-mediated interaction and CK2-dependent phosphorylation of syndapin I/PACSIN 1 drive InsP_6_-induced β cell endocytosis [6, 9, 12, 23, 36, 44, 45]. **E** Inhibition of CK2 effectively blocks InsP_6_-induced β cell endocytosis. **F** Exogenously-expressed wtPCS1SH3 (wSH3) but not mtPCS1SH3 (mSH3) competes with the SH3 domain of syndapin I/PACSIN 1 for dynamin I [23] resulting in dissociation of endogenous syndapin I/PACSIN 1 from dynamin I and inhibition of InsP_6_-induced β cell endocytosis. Arp2/3 (Arp2/3 complex), CK2 (casein kinase 2), D (dynamin), F-BAR (α-helical region of syndapin I/PACSIN 1), InsP_6_ (inositol hexakisphosphate), mSH3 (overexpressed mutant SH3), N-WASP (neural Wiskott–Aldrich syndrome protein), P (phosphoryl group), SH3 (Src homology 3 domain of syndapin I/PACSIN 1), SJ (synaptojanin) and wSH3 (overexpressed wild-type SH3)

The molecular machinery regulating exocytosis in the pancreatic β cell is quite well understood whereas the information regarding endocytosis is scarce. In the present study, we show for the first time that pancreatic β cells as well as insulin-secreting cell lines express syndapin I/PACSIN 1. This is interesting since this protein has previously been considered to be exclusively present in neurons [8, 9]. We could also demonstrate that syndapin I/PACSIN 1 is situated in abundant granule-like structures in the cytoplasm, but very rarely localizes in insulin-containing granules. These syndapin I/PACSIN 1-positive vesicles are smaller than insulin-containing granules and appear to be endocytic, endosomal or lysosomal vesicles. However, the identity of the subcellular organelles where syndapin I/PACSIN 1 resides remains to be clarified. Importantly, it is clear from the present work that syndapin I/PACSIN 1 knockdown impairs β cell accumulation of the endocytosis marker FM 1–43, following stimulation with glyceraldehyde/glucose or K^+^ depolarization. Hence, syndapin I/PACSIN 1 mediates β cells endocytosis (Fig. 7). These findings are relevant and add to our understanding of the details involved in the complex molecular machinery regulating pancreatic β cell endocytosis.

To further pinpoint the physiological importance of syndapin I/PACSIN 1 in β cell endocytosis, we evaluated the effect of syndapin I/PACSIN 1 knockdown on the presence of integral membrane proteins, here depicted as the density of L-type Ca^2+^ channels in the β cell plasma membrane. Voltage-gated L-type Ca^2+^ channels were chosen as representatives of the integral membrane proteins because of their ultimate importance for β cell function and survival [15, 22, 27]. Adequate numbers of L-type Ca^2+^ channels in the β cell plasma membrane mediate appropriate Ca^2+^ influx to meet requirements for Ca^2+^-dependent processes under a diverse range of physiological scenarios from insulin secretion to β cell survival [15, 22, 27]. Inappropriate increases or decreases of L-type Ca^2+^ channel density in the β cell plasma membrane inevitably result in intracellular Ca^2+^ overload or deficiency and consequently β cell dysfunction and even destruction [15, 22, 27]. Plasma membrane proteins, including β cell L-type Ca^2+^ channels, critically rely on the endocytic machinery for their internalization to exquisitely control their density in the plasma membrane [1–3]. Our findings demonstrate that syndapin I/PACSIN 1 regulates the density of L-type Ca^2+^ channels by endocytosis without influencing the conductivity of individual L-type Ca^2+^ channels. This is because syndapin I/PACSIN1 physically interacts with dynamin, N-WASP and other endocytotic proteins in the cytoplasm to mediate the internalization of pieces of the plasma membrane where some L-type Ca^2+^ channels reside, but not with L-type Ca^2+^ channels per se in the plasma membrane. This suggests that syndapin I/PACSIN 1 preserves the homeostasis of β cell plasma membrane proteins through endocytosis.

The most important mission of the present work was to clarify how syndapin I/PACSIN 1 mechanistically mediates InsP_6_-induced/dynamin I-dependent endocytosis. We now demonstrate that syndapin I/PACSIN 1 undergoes phosphorylation by InsP_6_-activated CK2 and subsequent interaction with N-WASP, enabling the syndapin I/PACSIN 1 SH3 domain-mediated orchestration of this endocytic event (Fig. 7). Thereby, the syndapin I/PACSIN 1 interaction partners N-WASP and dynamin I work in concert to play their individual roles (Fig. 7A–C). For example, the former serves to regulate actin cytoskeleton dynamics to remove local barriers to endocytic vesicle formation and the latter forms a collar around the neck of endocytic vesicles and drives their scission via GTP hydrolysis (Fig. A, B) [12, 33–37]. Syndapin I/PACSIN 1 can do so because syndapin I/PACSIN 1 transits between a closed and an open conformation at least under in vitro conditions (Fig. 7D–F) [38, 39]. In this context, the SH3 domain binds to the F-BAR domain to fold syndapin I/PACSIN 1 into a closed conformation that is considered an inactive form (Fig. 7D–F) [38, 39]. The interaction of the syndapin I/PACSIN 1 SH3 domain with the proline-rich motif of dynamin I releases the F-BAR domain from the F-BAR-SH3 clamp, thereby unfolding syndapin I/PACSIN 1 into an open conformation that is regarded active in dynamin I-dependent endocytosis (Fig. 7D, F) [38, 39]. Importantly, the conformational transition of syndapin I/PACSIN 1 from an inactive to an active form must be controlled by hitherto unknown signal(s). Interestingly, our present data suggest that in the β cell InsP_6_ serves as a priming signal for the transition of syndapin I/PACSIN 1 from closed to open conformation by activating CK2 induced phosphorylation of the protein (Fig. 7D–F). Incorporation of negatively charged phosphoryl groups into syndapin I/PACSIN 1 renders the F-BAR-SH3 clamp loose. This enables the loose SH3 domain to interact with the proline-rich motif of its interaction partners, such as dynamin I and N-WASP initiating dynamin I-dependent β cell endocytosis, according to the below sequence of events [9, 12, 40, 41]. First, it recruits dynamin I and N-WASP to endocytic sites and increases activities of N-WASP and dynamin I to rearrange cortical actin cytoskeleton and to pinch off endocytic vesicles, respectively (Fig. 7A, B) [9, 32, 42, 43]. Second, it releases homodimeric F-BAR module whose positively charged surface interacts with phospholipid membranes and drive membrane bending and subsequent endocytic steps (Fig. 7A, B) [38, 39]. Finally, it orchestrates complex interconnections among endocytic molecules, such as N-WASP and dynamin I (Fig. 7A, B) [38].

Overall, our findings demonstrate that syndapin I/PACSIN 1 acts as a player in β cell endocytosis. Thus, this protein has a role in preserving β cell plasma membrane homeostasis by regulating endocytosis of integral membrane proteins. Mechanistically, syndapin I/PACSIN 1, downstream of intracellular InsP_6_, serves as a substrate of InsP_6_-activated CK2. The resultant phosphorylation could conceivably prime the syndapin I/PACSIN 1 SH3 domain with the capability to interact with the endocytic players dynamin I and N-WASP for executing β cell endocytosis.

## Supplementary Information

Below is the link to the electronic supplementary material.

Supplementary file1 (DOC 25 KB)

Supplementary file2 (TIF 88507 KB)

Supplementary file3 (TIF 59550 KB)

## Data Availability

The data that support the findings of this study are available from the corresponding author upon reasonable request.
