# Supplementary material for: Inositol hexakisphosphate primes syndapin I/PACSIN 1 activation in endocytosis
Source: Cell Mol Life Sci. 2022 May 9;79(6):286. doi: 10.1007/s00018-022-04305-2 (PMC9085685; doi:10.1007/s00018-022-04305-2)
Supplement: Supplementary file 1 — Supplementary file1 (DOC 25 KB) [file 18_2022_4305_MOESM1_ESM.doc]

**Supplementary figure legends**

**Supplementary Fig. 1** Immunocytochemical characterization of guinea pig polyclonal antibody against insulin in rodent islets. Confocal images of insulin immunofluorescence in mouse (upper) and rat islets (lower) exposed to the primary anti-insulin antibody preabsorbed with bovine insulin (left), to the primary antibody-omitted solution (middle) or to the primary antibody alone. Bars = 10 μm. The experiments were repeated four times.

**Supplementary Fig. 2** Immunocytochemical characterization of rabbit polyclonal antibody against syndapin I/PACSIN 1 in rodent islets. Confocal images of syndapin I/PACSIN 1 immunofluorescence in mouse (upper) and rat islets (lower) incubated with (right) and without the primary anti-syndapin I/PACSIN 1 antibody (left). Bars = 10 μm. The experiments were repeated four times.
