# Supplementary figures and images for: Inositol hexakisphosphate primes syndapin I/PACSIN 1 activation in endocytosis
Source: Cell Mol Life Sci. 2022 May 9;79(6):286. doi: 10.1007/s00018-022-04305-2 (PMC9085685; doi:10.1007/s00018-022-04305-2)

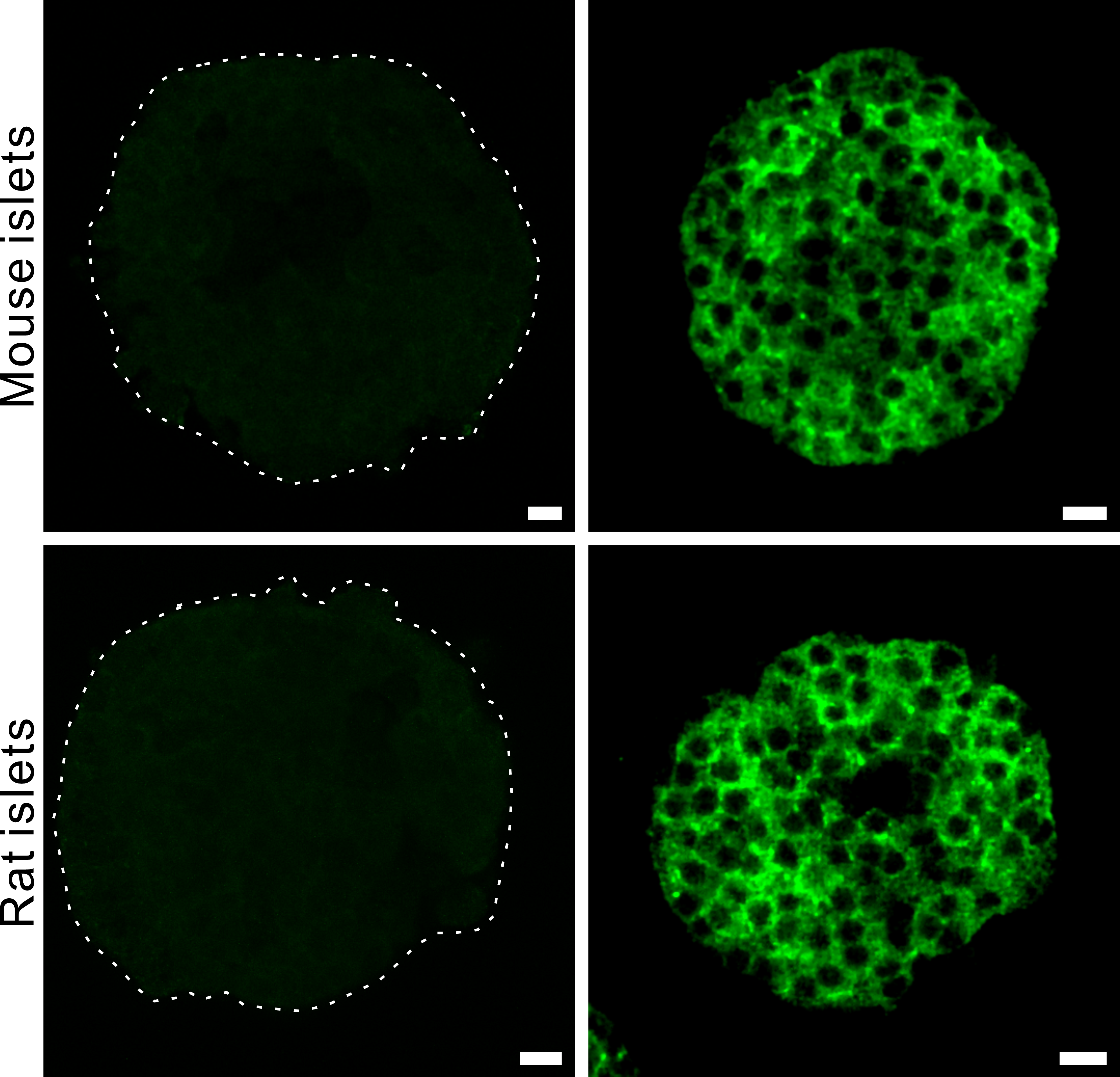

Supplement: Supplementary file 3 — Supplementary file3 (TIF 59550 KB) [file 18_2022_4305_MOESM3_ESM.tif]
